# The nutritional quality of the meals and foods provided to beneficiaries of the Brazilian Worker’s Food Program: a systematic review

**DOI:** 10.1017/jns.2025.20

**Published:** 2025-03-22

**Authors:** Fernanda Martins de Albuquerque, Nathália César Nunes, Vanessa Manfre Garcia de Souza, Cintia Chaves Curioni, Daniel Henrique Bandoni, Daniela Silva Canella

**Affiliations:** 1 Instituto de Nutrição, Universidade do Estado do Rio de Janeiro/UERJ, Rua Francisco Xavier, 524, Maracanã, Rio de Janeiro, Brazil; 2 Instituto de Saúde e Sociedade, Universidade Federal de São Paulo/UNIFESP, Rua Silva Jardim, 136, Vila Matias, Santos, SP, Brazil

**Keywords:** Diet, Food Service, Nutrition Policy, Obesity, Workplace

## Abstract

The Brazilian Worker’s Food Program (WFP) is a public policy initiative that focuses on nutritional assistance for low-income formal workers (less than five minimum wages). Currently, it serves more than 25 million formal workers (around 54%). This systematic review aimed to assess the nutritional quality of meals offered and/or consumed by beneficiaries of the WFP. Observational studies conducted with workers from companies registered in the programme were eligible, with no restrictions on the period of publication. The nutritional quality was assessed according to the guidelines of the programme (Normative Ordinance No. 66/2006). Twenty cross-sectional studies and one cohort study met the inclusion criteria. Most of the participants were male, from manufacturing industries, and their average age was 35.0 years. The results of the analysis showed that fibre, sodium, calories, and proteins were the nutrients that most exceeded the recommended amounts, whereas carbohydrate was the nutrient that had the least amount. The results showed that the nutritional quality of the food offered to or consumed by workers did not fully meet the required guidelines and, in some companies, did not promote an adequate and healthy diet. The WFP has great potential and needs to be reformulated to make it a programme that contributes to strengthening the realisation of the human right to adequate food.

The issue of workers’ food and nutrition has mobilised organisations and countries around the world.^(1–3)^ Ensuring a well-nourished and healthy workforce is a critical component of social protection measures for workers, yet it remains absent from initiatives aimed at enhancing working conditions and occupational safety and health, particularly for informal workers.^(4)^ As food and nutrition are acknowledged as human rights, the government is responsible for the development of food and nutrition security policies aimed at strengthening the realisation of human rights.^(5)^

The workplace is considered strategic for promoting adequate and healthy food as well as for reducing disparities in food access.^(6)^ However, workers can be exposed to an obesogenic environment, both inside and outside the workplace, especially in the form of receiving benefits such as vouchers, when they purchase food or meals in food establishments that are limited to healthy options.^(7,8)^ In this context, workers are influenced by an environment that encourages the consumption of ultra-processed foods and beverages, potentially resulting in decreased productivity and an upsurge in the prevalence of noncommunicable diseases, carrying significant global economic ramifications.^(9,10)^

The Workers’ Food Program (WFP), established in 1976 in Brazil, has become an important public policy with voluntary adhesion by companies, being targeted at the formal working adult population.^(11)^ In 2024, the WFP provides support to 308,335 registered companies and more than 25 million employees nationwide. The WFP is a government initiative that uses tax incentives to encourage employers to provide adequate food to their employees, prioritising workers who receive up to five minimum wages but are extensible to workers with income above this limit.^(12)^ Its coverage has gradually evolved, increasing the number of registered small and medium-sized companies and, consequently, benefiting workers.^(13,14)^ In Brazil, this initiative stands as the primary public policy targeting the formal workforce. Companies that adhere to the WFP can determine ways to provide meals or food for workers in the workplace or hire an outsourced company for the service. In this case, both the contracting and outsourced companies must be registered in the programme and have a technical nutritionist responsible for menu planning. Outsourced options such as prepared meals, meal vouchers, food vouchers, and food baskets are allowed. These ways to provide meals or food for workers are defined in the legislation as modalities.^(15)^

More than one modality of benefit may be adopted, and the same worker may receive two or more benefits of different types. Most workers received food vouchers (40.0%) and meal vouchers (24.0%), followed by meals produced by third-party services (kitchen management or transported meals) (21.3%), food baskets (9.3%), and meals at the workplace (5.3%).^(16)^ The beneficiary company joins the WFP by registering using the electronic forms available on the Ministry of Labor’s website. Enrolment and registration in the programme are valid immediately and for an indefinite period and may be completed at the initiative of the enrolee or registrant, for whatever reason. To stay on the programme, companies must comply with nutritional composition criteria set out in specific legislation for the supply of food and meals to workers.^(17)^ The different modalities contribute to greater coverage, considering the companies’ characteristics and possibilities; however, some of them can lead to difficulties in the implementation of nutritional guidelines.^(18)^

Throughout almost 50 years of the existence of the WFP, studies have been developed to assess the political aspects, the nutritional composition of the supplied meals, and its relationship to workers’ health;^(19–22)^ however, there is a lack of a national studies to assess the programme’s implementation and its impact or at least a systematisation of the results of the studies. Given the potential of the WFP to ensure food and nutritional security, it is crucial to understand its current status and implementation methods, especially in the context of nutritional recommendations (henceforth called guidelines) of Normative Brazilian Ordinance No. 66/2006^(15)^ — the regulation of the composition and distribution of calories and nutrients and food groups to be offered in meals, based on the WHO’s Global Strategy on Healthy Eating, Physical Activity and Health^(23)^ and the 1st edition of the ‘Brazilian Dietary Guidelines’^(24)^ — have been absent since 2021.^(25–27)^ Despite its extensive history and numerous legislative and regulatory changes, no study has systematically gathered evidence on the operationalisation and nutritional quality of meals or food provided across all modalities. This study aimed to conduct a systematic review of the available evidence regarding the nutritional quality of the meals and food provided and/or consumed by beneficiaries of the WFP.

## Methods

The systematic review was previously registered in PROSPERO (registration number: CRD42023441996) and was reported based on the recommendations of the Preferred Reporting Items for Systematic Reviews and Meta-Analyses (PRISMA) guidelines.^(28)^

The review was designed to address the following research question: ‘Do the meals and/or foods offered and/or consumed by Brazilian WFP beneficiaries have the nutritional quality recommended by the programme?’ The research question was defined based on the strategy Population, Exposure, Comparators, Outcomes, and Study designs (PECOS)^(29)^ presented in Table 1.

**Table 1.** Definition of Population, Exposure, Comparators, Outcomes, and Study Designs (PECOS) for the systematic review, 2025

P	Population of interest or topic to be addressed	Workers from companies registered with the Brazilian WFP
E	Exposure	Brazilian WFP
C	Comparison group reported	Not applicable (specific to randomised controlled trials)
O	Outcome	Nutritional quality (food groups or nutrient composition of the food offered and/or consumed)
S	Study design	Observational studies that assessed the Brazilian WFP

WFP, Worker’s Food Program.

### Eligibility criteria

The inclusion criteria, based on the acronym (PECOS), were as follows: Observational studies (cross-sectional, cohort, case-control) that evaluated the nutritional quality of the food offered (assessed by different methodologies such as analysis of menus/per capita/portioning/weighing meals) and/or consumed (evaluated by food consumption surveys, such as a 24-h dietary recall) by the population of economically active workers from companies registered in the Brazilian WFP. The evaluation must consider aspects of the nutritional guidelines of the Normative Brazilian Ordinance No. 66/2006^(15)^ such as calories, carbohydrates, proteins, total fat, saturated fat, sodium, and fibre, the offering of fruits and vegetables, NDPCal (Net Dietary Protein Calorie), or other methodologies to assess the overall quality of meals (such as energy density, *a priori* pattern evaluation, such as meal quality index, based on food groups or nutrient composition, or other indexes), with any different modalities, in which the company can provide (1) meals at the workplace, prepared in self-management or by third-party services; (2) meal or food vouchers, tickets, or coupons to be used is an under agreement establishments network; or (3) the food baskets.

Studies that assessed meals offered for individuals with pre-existing medical conditions or specific diet conditions, such as allergies and intolerances; inborn errors of metabolism; pathologies that restrict the consumption of certain foods, such as celiac disease, diabetes, kidney disease, dyslipidaemia, and hypertension; pregnant and lactating women; vegans and vegetarians; papers that did not meet the inclusion criteria (nutritional quality evaluation of the food offered and/or consumed by Brazilian WFP beneficiaries); and studies that did not evaluate compliance with nutritional guidelines according to the Ordinance No. 66/2006^(15)^; intervention studies; animal or in vitro experiments; qualitative studies; review articles; book sections; conference proceedings; editorials; letters to the editor; response letters; notes; comments; and publications for which the abstract and full text were unavailable were excluded.

### Study identification and selection

In October 2024, a comprehensive literature search was performed using the following electronic databases: PubMed, Lilacs, Embase, Web of Science, Scopus, Google Scholar, and the *Coordenação de Aperfeiçoamento de Pessoal de Nível Superior* (CAPES) catalogue of theses and dissertations. A combination of descriptors available at the Medical Subject Headings (MeSH) index and keywords consistently referred to in papers related to the issue was used (see Supplementary Material 1 for the search strategy carried out in each database). There were no restrictions on language or period of publication. Additional studies were identified by searching the references of the papers included in this review.

We exported the results to the software EndNote® Web to check for duplicates. Two reviewers (F.M.A. and N.C.N.) independently searched for the papers, reviewed the titles and abstracts, and performed a full reading of the studies. In instances of discrepancies, the two researchers deliberated to achieve consensus or, when deemed essential, sought the perspective of a third researcher. (D.S.C.). The selection of the papers (both in the title/abstract screening and the full-text screening) was performed using the Rayyan® tool.

A pilot test was carried out with the Rayyan® tool before starting the selection by the review team. The pilot test included (1) the selection of the first 20 titles (abstracts) in alphabetical order, (2) screening by the researchers using the eligibility criteria, and (3) discussion of discrepancies by the researchers about the eligibility criteria. According to Fleiss’s kappa statistics, the team started the screening only when 75% (or more) of the searches agreed.

### Assessment of the methodological quality of studies

Two reviewers independently assessed the methodological quality of the studies (F.M.A. and N.C.N.) using the critical appraisal tools of observational studies from the Joanna Briggs Institute.^(30)^ For cross-sectional studies, the tool included eight questions addressing the domains of eligibility, confounding factors (criteria considered not applicable when the study was descriptive in nature), measurement of exposure, and outcome and analyses (considered appropriate if it described the statistical analyses performed, the use of normality tests, the statistical analysis software and the level of statistical significance used). For cohort studies, the tool consisted of eleven questions addressing the domains of eligibility, confounding factors, measurement of exposure and outcome, follow-up time, losses, and analyses. For each domain, the two reviewers independently answered ‘yes’, ‘no’, ‘unclear’, or ‘not applicable’. The percentage of ‘yes’ answers to each study was calculated. Any discrepancies were resolved through discussion or by the nomination of a third reviewer (D.S.C.).

### Data extraction and synthesis

The data were extracted using a standardised form developed by two reviewers (F.M.A. and N.C.N.) using Google Forms®. This instrument was previously tested in a random sample of five articles and refined after being checked by the other authors to ensure that all relevant information was retrieved. Each reviewer extracted data from the articles, and their work was carefully checked by another reviewer. Disagreements were resolved between the authors in charge of data extraction, and if necessary, a third author (D.S.C.) was involved.

The following information was extracted: authors and year of publication; year of data collection; location of the study; number of participating companies/workers, comparison of workers from companies adherent and non-adherent to the WFP, age, sex, purpose of the study, study methodology, study duration (if applicable), WFP modality, the presence of nutritionists who were technically responsible, quality and quantity of foods/nutrients offered/consumed, amount of calories, energy density, summary indicator of meal quality, food/meal assessment and related recommended amounts, other relevant results of the study (related to health outcomes/anthropometrics of workers), and study limitations reported by the authors.

To analyse whether the results observed were in line with the normative structure of the programme,^(15)^ the results were summarised by applying a multidimensional methodology based on structure (obtained through research of the programme’s legislation) — process (analysis of the programme’s operationalisation) — outcome (whether the results obtained are those proposed in the structure), which was previously used to assess the programme in the period to 1995–2002,^(22)^ before the implementation of Normative Brazilian Ordinance No. 66/2006.^(15)^ Conceptual models increase the efficiency/effectiveness of research, generalisation, and interpretation of research findings. By specifying factors that have been shown in previous research to influence the phenomenon of interest, in this case, the implementation of a policy to benefit worker’s health and nutrition, conceptual frameworks increase the relevance of research and conclusions to inform implementation practices.^(31)^ Additionally, a critical and qualitative assessments were made based on the results of the studies.

## Results

### Description and general findings of the included studies

**Fig. 1.**
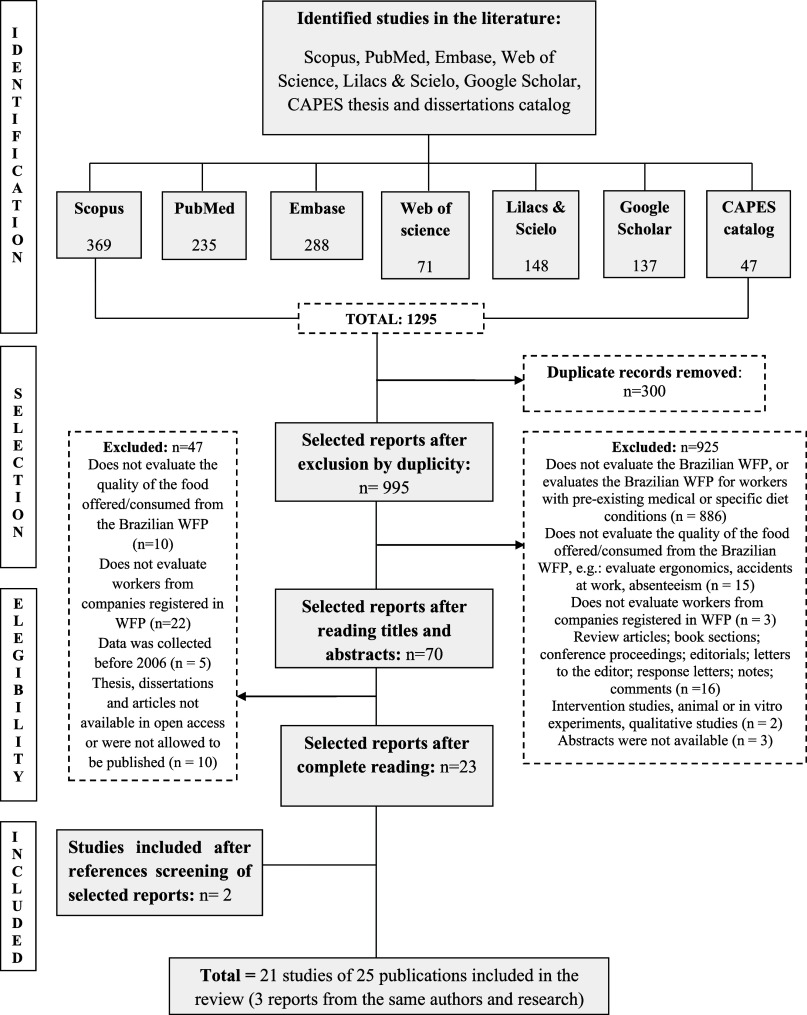
PRISMA flow diagram for identification and selection of studies eligible for the systematic review, 2025.

Initially, we identified 1295 records from the databases. After removing duplicate publications, a total of 995 articles remained. After evaluating titles and abstracts, sixty publications were evaluated in full. The authors of the ten unavailable publications were contacted, but no replies were received. Finally, twenty-five publications were included in this review. The process of identification and selection of studies eligible for the systematic review is shown in Fig. 1.

Table 2 presents the characteristics of the studies included. Twenty cross-sectional studies^(32–51)^ and one cohort study^(52)^ were included. These studies were conducted between 2006 and 2022. The number of companies evaluated among the studies ranged from 1 to 40, and the number of workers included ranged from 9 to 1480. Most participants were male and aged between 18 and 50 years. The studies were carried out in ten states distributed across three of the five Brazilian regions. Regarding company types, studies were carried out in manufacturing industries, services, and trade (n = 2),^(33,36)^ manufacturing industries (n = 13),^(32,34,37,38,41–44,48–52)^ and services and trade (n = 4).^(40,45–47)^ Most of the workers had completed high school, had an income of less than five minimum wages (1 minimum wage = R$ 1.412,00 or $258,54 in 2024), which is aligned with programme criteria (data not shown in the table).

**Table 2.** Characteristics of the studies included in the systematic review, 2025

Author (year)/ Place of study	Study design	Number of companies	Number of workers	Age (average in years)	Sex	Year of data collection	Assessed outcomes	Methods	WFP modality evaluated	Presence of a technical responsible nutritionist
Previdelli et al. (2010)/ São Paulo/São Paulo	Cross-sectional	1	202	33.4 ± 9.4	51% F 49% M	2006	Food consumption. Nutritional status, physical activity. Meal quality of consumed meals by Health Eating Index revised.	24-h recall	NI	NE
Canella et al. (2011)/ São Paulo/São Paulo	Cross-sectional	21	NI	NI	NI	2006/2007	Meal quality of offered meals by energy density.	Analysis of menus and per capita (3 d)	Self-management	Yes
Mesquita & Mesquita (2013)/ Fortaleza/Ceará	Cross-sectional	2	103	35.1	77% F 23% M	2011	Food offered. Nutritional status, Health conditions.	Analysis of menus and per capita (5 d)	Self-management	NE
Lanci & Matsumoto (2013)/Paranavaí/Paraná	Cross-sectional	1	99	32.0	28% F 72% M	2009	Food offered. Nutritional status.	Analysis of menus (3 d)	Self-management	Yes
Pereira et al. (2014)/ Santos/São Paulo	Cross-sectional	4	1480	NI	NI	2012	Meal quality of offered meals by qualitative assessment of menu preparations.	Analysis of menus and per capita (3 d)	Self-management	Yes
Cunha & Barbosa (2014)/Itaboraí/Rio de Janeiro	Cross-sectional	2	150	NI	13% F 87% M	2011/2012	Food offered by analysis of menus.	Evaluation of portioning in meal distribution	NI	NE
Duarte et al. (2015) -	Cross-sectional	1	380	NI	NI	2013	Food offered by analysis of menus (3 d). Meal quality index of offered meals.	Weighing meals	Self-management	Yes
Batista et al. (2015)/Curitiba/Paraná	Cross-sectional	1	19	30.0	32% F 68% M	2014	Food offered. Nutritional status, health conditions, smoking, physical activity, alcohol consumption.	Analysis of menus and per capita (20 d)	Self-Management	Yes
Bezerra (2015) e Bezerra et al. (2017)/ Rio Grande do Norte	Cross-sectional	26 (13 WFP e 13 non-WFP)	1069 WFP (541) e non-WFP (528)	34.5	54.52% M (WFP) 64.70% M (non-WFP)	2014	Food consumption. Nutritional status, Health conditions, physical activity, smoking or alcohol consumption.	Anthropometric evaluation and 24-h recall	Self-Management	Yes
Oro & Hautrive (2016)/ São Miguel do Oeste/Santa Catarina	Cross-sectional	1	300	NI	NI	NI	Food offered.	Analysis of menus and per capita (10 d)	NI	NE
Ferreira (2016)/São Luís/Maranhão	Cross-sectional	1	9	NI	100% M	2015	Consumption of the meals offered.	Evaluation of portioning in meal distribution (3 d)	Transported meal (third-party services)	Yes
Bortolozo et al. (2016)/ Ponta Grossa/Paraná	Cross-sectional	NA	292 (87 workers in the administrative sector and 205 in the production sector)	32.6 ± 8.84	23%F 77% M	NI	Consumption of the meals offered.	Weighing meals	Self-management	NE
Salvetti & Possa (2017)/Região sul	Cross-sectional	1	150	NI	NI	2014	Food offered.	Analysis of menus (71 d)	Self-management	Yes
Hartmann et al. (2019)/Marau/Rio Grande do Sul	Cross-sectional	1	37	39.4 ± 9.3	75.7%F	NI	Food consumption. Nutritional status.	‘How is your diet?’ Test	NI	NE
Albuquerque & Lima (2020)/Maceió/Alagoas	Cross-sectional	1	80	NI	NI	2019	Food offered.	Menu analysis based on technical sheets (7 d)	Self-Management	NE
Guilherme et al. (2020)/Recife/Pernambuco	Cross-sectional	40	NI	NI	NI	2014/2015	Meal quality of offered meals by qualitative assessment of menu preparations.	Analysis of menus (30 d)	Self-Management	Yes
Torres et al. (2020)/Rio Grande do Norte	Cohort	16	273 (143 WFP e 130 non-WFP)	37.2	55.2% M (WFP) 50.8% M (non-WFP)	2014	Food consumption. Nutritional status, Consumption of the meals offered, physical activity.	Anthropometric evaluation and 24-h recall	NI	NE
Souza (2020)/Rio Grande do Norte	Cross-sectional	33	921	38.3	44.1% F 55.8% M	2017/2018	Food consumption. Nutritional status, Consumption of the meals offered, Motivations for food choice.	Anthropometric evaluation and 24-h recall and food choice questionnaire	NI	NE
Costa et al. (2022)/Rio Grande do Norte	Cross-sectional	33	929 (445 WFP e 484 non-WFP)	38.6	NI	2017/2018	Food consumption. Nutritional status, Consumption of the meals offered, physical activity.	Anthropometric evaluation and 24-h recall	NI	NE
Bergamin (2022)/Brusque/Santa Catarina	Cross-sectional	1	320	NI	NI	2022	Food offered.	Analysis of menus and per capita (5 d)	Self-management	NE

NE, not evaluated; NI, not informed; M, male; F, female; R24h, 24-h recall; WFP, Worker’s Food Program.

Most of the methods used in the selected studies assessed food offered to workers by the menu evaluation (n = 10),^(33–36,40,41,44,45,49,51)^ mean quantity of food per capita planned to be offer (n = 6),^(33,34,36,41,45,49)^ evaluation of portioning in the distribution of meals (n = 2),^(46,47)^ and weighing meals (n = 2).^(48,50)^ Additionally, some studies focused on the food consumed by workers, using methods for the evaluation of individual food consumption such as 24-h dietary recall (n = 7),^(32,37–39,42,43,52)^ and anthropometric assessment as a proxy for the impact of meal quality of WFP (n = 5)^(37,38,42,43,52)^ (Table 2). The methods used to assess food consumption were the 24-h recall (n = 6),^(32,37,38,42,43,52)^ ‘How is your diet?’ test (n = 1)^(39)^ and FFQ (n = 1)^(37)^ (Table 2). Lunch was the most evaluated meal by studies, ranging from 3 to 71 d of menu evaluation.^(33–38,40,42–52)^

### Structure dimension of programme’s legislation

This dimension is obtained through research of the programme’s legislation. Throughout its history, three ordinances have regulated the nutritional guidelines of the WFP. The first, Ordinance No. 652, on 22 December 1976,^(53)^ determined that the main meals (lunch, dinner, and supper) should offer at least 1400 kcal; breakfast and snack, at least 300 kcal; and a protein-calorie percentage (NDPCal%) of meals of at least 6%. The Interministerial Ordinance No. 5/99^(54)^ allows a reduction to 1,200 kcal in the case of light activity or an increase to 1,600 kcal in the case of intense activity, for large meals, subject to technical justification. Only in 2006^(15)^ were the guidelines no longer restricted to the energy and protein intake of meals. At this time, in addition to the reduction in the energy value of the main meals (which became 600 kcal to 800 kcal), with the possibility of an increase, the distribution of nutrients in the meals (macronutrients, fibre, and sodium) is provided for, in addition to the daily offer of fruit, vegetables, and greens. In the following years, there were no further updates to the WFP. In 2021, Decree No. 10.854 changed its regulations, formally revoking Interministerial Ordinance No. 66/2006, which determined the nutritional guidelines of the programme, and established that ‘It is up to the Ministry of Health and the Ministry of Labor and Social Security to jointly regulate aspects related to health promotion and food and nutritional security of the WFP’.^(25)^

### Process dimension of the programme’s operationalisation in the workplace

The process dimension included an analysis of the operationalisation of the programme, observing the positive and negative aspects resulting from its implementation.

Most of the companies evaluated had self-management as a modality for implementing the WFP^(33–36,38,40,41,43–45,48,50,51)^ (Table 2). This type of operation includes a proposal in the programme’s structure, in which the employer is responsible for selecting and purchasing the foodstuffs to be prepared and served to the workers.

Of the twenty-one studies, only ten reported having a nutritionist as the Technical Responsible^(33,35,36,38,43–45,47,48,51)^(Table 2). In a study that assessed twenty-one companies, only fifteen had technical supervision by a nutritionist, whose menu had a lower median energy density.^(33)^ It is important to note that the presence of this professional is essential to ensure that the programme operates as it has been structured, to generate expected results.

According to the data from interviews with forty nutritionists, none of them related the WFP to food and nutrition education actions, and when asked about the motivations for companies to join the programme, most of the nutritionists replied that it was the tax benefit that the company would receive.^(51)^ The superficial knowledge of nutritionists and lack of awareness of managers can lead to inadequate performance in relation to the legislation, failing to take on key responsibilities for the proper WFP implementation.

### Results dimension of nutritional quality of the meals and foods

The results dimension dialogues with all others, considering whether the results obtained are those proposed in the structure, and if so, the explanatory factors are to be found in the programme structure or the process.

Among the studies selected for this analysis, twelve assessed the adequacy of calories and the distribution of the nutrient percentage of lunch offered to workers according to Normative Ordinance No. 66/2006.^(34–36,40,41,44–50)^ The findings indicated that carbohydrate was the nutrient that least reached the minimum amount according to the nutritional guidelines, falling short of the recommended standards,^(34,36,41,44–46,48,49)^ with a maximum of 35.5% inadequacy^(34)^ and a minimum of 3.3%.^(46)^ Conversely, some nutrients exceeded the guidelines, such as proteins^(34,36,40,41,44–50)^ with a maximum of 36.4% inadequacy^(34)^ and a minimum of 3%,^(46)^ calories^(34–36,40,41,44–48)^ with a maximum of 937.7 calories (217.2%) exceeded^(36)^ and a minimum of 58.4 calories (107.3%),^(40)^ sodium^(36,40,41,44–46,48,50)^ with a maximum of 2340.2 mg (343.7%) exceeded^(36)^ and a minimum of 40 mg (104.2%),^(40)^ and fibre^(36,40,41,44–46,48,50)^ with a maximum of 13.1 g (231%) exceeded^(46)^ and a minimum of 2.3 g (123%).^(41)^ Saturated fat^(36,41,44–46,50)^ showed the greatest adequacy (Table 3).

**Table 3.** Compliance with the nutritional guidelines according to Normative Ordinance No. 6/2006, 2025

Energy and nutrients	Values for meals	Do not reach	Reach	Exceeds
Total energy value (kcal)	Lunch/dinner/supper: 600–800 Breakfast/snack: 300–400	–	Oro & Hautrive, 2016; Bortolozo et al., 2016	Mesquita & Mesquita, 2013; Lanci & Matsumoto, 2013; Pereira et al., 2014; Cunha & Barbosa, 2014; Duarte et al., 2015; Batista et al., 2015; Ferreira, 2016; Salvetti & Possa, 2017; Albuquerque & Lima, 2020; Bergamin, 2022
Carbohydrates (%)	Lunch/dinner/supper/breakfast/snack: 60	Mesquita & Mesquita, 2013; Pereira et al., 2014; Cunha & Barbosa, 2014; Duarte et al., 2015; Batista et al., 2015; Oro & Hautrive, 2016; Salvetti & Possa, 2017; Bergamin, 2022	Lanci & Matsumoto, 2013; Bortolozo et al., 2016	Ferreira, 2016; Albuquerque & Lima, 2020
Protein (%)	Lunch/dinner/supper/breakfast/snack: 15	–	–	Mesquita & Mesquita, 2013; Pereira et al., 2014; Cunha & Barbosa, 2014; Duarte et al., 2015; Batista et al., 2015; Oro & Hautrive, 2016; Ferreira, 2016; Bortolozo et al., 2016; Salvetti & Possa, 2017; Albuquerque & Lima, 2020; Bergamin, 2022
Total fat (%)	Lunch/dinner/supper/breakfast/snack: 25	Oro & Hautrive, 2016; Salvetti & Possa, 2017; Albuquerque & Lima, 2020	Mesquita & Mesquita, 2013; Lanci & Matsumoto, 2013; Cunha & Barbosa, 2014; Bortolozo et al., 2016	Pereira et al., 2014; Duarte et al., 2015; Batista et al., 2015; Ferreira, 2016; Bergamin, 2022
Energy and nutrients	Values for meals			
		Do not reach	Reach	Exceeds
Saturated fat (%)	Lunch/dinner/supper/breakfast/snack: <10	Albuquerque & Lima, 2020	Pereira et al., 2014; Cunha & Barbosa, 2014; Batista et al., 2015; Bortolozo et al., 2016; Salvetti & Possa, 2017; Bergamin, 2022	Lanci & Matsumoto, 2013; Duarte et al., 2015
Fibre (g)	Lunch/dinner/supper: 7–10 Breakfast/snack: 4–5	Oro & Hautrive, 2016	–	Pereira et al., 2014; Cunha & Barbosa, 2014; Duarte et al., 2015; Batista et al., 2015; Bortolozo et al., 2016; Salvetti & Possa, 2017; Albuquerque & Lima, 2020; Bergamin, 2022
Sodium (mg)	Lunch/dinner/supper: 720–960 Breakfast/snack: 360–480	Oro & Hautrive, 2016	–	Pereira et al., 2014; Cunha & Barbosa, 2014; Duarte et al., 2015; Batista et al., 2015; Bortolozo et al., 2016; Salvetti & Possa, 2017; Albuquerque & Lima, 2020; Bergamin, 2022
NDPCal (%)^*^	Lunch/dinner/supper/breakfast/snack: 6–10	–	–	–

* Protein-calorie percentage.

Note: Considering total daily value for energy – 2000 kcal, carbohydrates – 55–75%, protein – 10–15%, total fat – 15.30%, saturated fat <10%, fibre – 25 g, sodium – 2400 mg, NDPCal – 6–10. All modalities must meet the same daily reference values.

Only a small number of studies (n = 5) utilised a summary indicator of the nutritional quality of meals offered to workers. The indicators used for this assessment were the Meal Quality Index (n = 2),^(36,48)^ the Qualitative Assessment of Menu Preparations (n = 2),^(36,51)^ the modified version of the Healthy Eating Index (n = 1),^(32)^ and energy density (n = 1)^(33)^ (Table 2). Generally, the indicators showed inadequacies, considering the nutritional parameters adopted by each one, such as the high intake of added salt (consumed by 99.8% of workers) and added sugar (white or brown) content (consumed by 93.2% of workers)^(37)^; higher terciles of energy density accompanied by lower levels of iron (1^st^ tercile 6.60 to 3^st^ 5.51 mg/1000 kcal) and fibre (1^st^ tercile 11.24 to 3^st^ 8.72 g/1000 kcal)^(33)^; and the high frequency of occurrence in the menu in the days evaluated of the fried foods (75%), fatty meats (50%), sweets (66.6%), foods rich in sulphur (50%), and repetition of colours (50%).^(36)^ The only study that evaluated food consumption using the NOVA classification, based on the extent and purpose of food processing, found that ultra-processed foods contributed 20.6% of the total energy in workers’ diets.^(37)^

Overall, regardless of the company’s type (industry, service, or trade), inadequacies were observed in relation to the WFP guidelines. Only one study has investigated meal quality indicators according to company type.^(33)^ The median value of energy density for services and trade was higher (1.58 kcal/g) than for industries (1.39 kcal/g).^(33)^ Moreover, one study compared the food consumption of workers in different sectors.^(50)^ Compared to administrative workers, the energy (+ 156.64 kcal), protein (+ 4.97 g), carbohydrate (+ 32.14 g), and fibre (+ 4.26 g) intakes of production workers were higher than those of administrative workers (data not shown in the table).

Some cross-sectional studies have assessed the nutritional status of workers as a proxy for the impact of meal quality on WFP. Ten studies evaluated the nutritional status of the participants (Table 2) using the BMI classification,^(34,38,42,43,50)^ waist circumference (WC),^(35,38,42,43)^ and energy balance^(50)^ as the main parameters by measuring energy expenditure and food consumption (data not shown in the table). In general, a high prevalence of overweight and obesity was observed among the study participants^(32,34,35,37,39,43,45,50)^ (data not shown in the table). Only one cohort study compared workers from manufacturing industries that were adherent and not adherent to the WFP, the change in indicators of nutritional status and food consumption at two evaluations with a 4-year interval, and showed that workers in WFP-adherent companies have an increase in BMI and WC over time, which is higher than that observed among workers in non-WFP-adherent companies.^(52)^

Considering the importance of WFP as a programme to promote healthy habits, other variables related to health and nutrition, such as physical activity level^(32,38,42,43,45,52)^ (n = 6), general health conditions (n = 4),^(34,38,43,45)^ smoking (n = 3),^(38,43,45)^ alcoholic beverage consumption (n = 3),^(38,43,45)^ and motivations for food choices (n = 1)^(37)^ (Table 2), were also analysed. These studies showed a highly sedentary lifestyle (which can represent a less healthy lifestyle and influence diet quality) among the workers evaluated.^(32,45)^ Regarding health conditions, it was observed that some participants had noncommunicable diseases.^(34,45)^

Additionally, in other analyses, four selected studies compared companies registered in the WFP with companies not registered^(38,42,43,52)^ aimed to assess the impact of WFP on workers’ nutritional status, given the growing prevalence of obesity throughout the world. In general, based on this comparison, studies have shown that WFP benefit workers have a higher education level and monthly income^(38,42,52)^ and specific training for the position held in the company^(52)^ (data not shown in the table). On the other hand, they also had a higher BMI^(38,42,43)^ and mean WC^(42,43,52)^ and thus were classified as having a greater prevalence of obesity and cardiovascular risk (Table 4). Regarding the assessment of food consumption, lower consumption of sodium and saturated fat was observed at lunch^(38,43)^ and at lunch and other meals^(38)^ by WFP benefit workers. Moreover, lower consumption of protein was observed at lunch and other meals by WFP benefit workers.^(38)^ When comparing women who benefited from WFP to those who did not, it was observed that WFP beneficiaries had lower sodium intake at lunch, other meals, and in the daily total.^(42)^ When comparing the benefits and non-benefits of WFP for men, there was a greater consumption of carbohydrates and fibre at lunch and a lower consumption of carbohydrates and fibre at other meals due to WFP^(42)^ (Table 4).

**Table 4.** Results of comparisons between workers who are beneficiaries and non-beneficiaries of the Worker’s Food Program, 2025

Author (year)	Food consumption (24-h recall)						
	Calories	Carbohydrates	Protein	Lipid	Saturated fat	Fibre	Sodium
	*WFP Group × non-WFP Group*						
	Lunch						
Bezerra (2015)	=	=	=	=	<	=	<
Bezerra et al. (2017)	=	=	=	=	<	=	<
Costa et al. (2022)	Female lunch						
	=	=	<	=	=	=	<
	Male lunch						
	=	>	=	=	=	>	=
Bezerra (2015)	Lunch + other meals						
	=	=	<	=	<	=	<
	Other meals						
	=	=	<	=	=	=	<
Costa et al. (2022)	Female other meals						
	=	<	=	=	=	=	<
	Male other meals						
	=	<	=	=	=	<	=
	Female daily total						
	<	<	=	<	=	=	<
Torres et al. (2020)	*WFP Group: year 2018 × 2014*						
	Daily total						
	=	=	>	=	=	=	=
	Anthropometric variables						
	BMI			AC		WC	
	*WFP Group × non-WFP Group*						
Bezerra (2015)	>			>		NE	
Bezerra et al. (2017)	>			NE		>	
	*WFP Group × non-WFP Group: year 2018 × 2014*						
Torres et al. (2020)	=			NE		>	
	*WFP Group × non-WFP Group: male*						
Costa et al. (2022)	>			NE		>	

NE, not evaluated; WFP, Worker’s Food Program; WC, waist circumference; AC, abdominal circumference.

The significance of ‘=’, ‘>’, and ‘<’ signals was ‘equal’, > ‘higher’, and < ‘lower’.

### Methodological quality of studies

Tables 5 and 6 present the methodological assessments of the included studies. Of the twenty cross-sectional studies, only six received ‘yes’ for all the domains evaluated. All cross-sectional studies received high ratings in the criteria used for the measurement of the condition, and the majority received high ratings for valid and reliable exposure measures. According to the checklist items, the main limitations include the fact that the criteria for inclusion in the sample were not clearly defined and that the study subjects and the setting were not described in detail. Low scores were also obtained for the outcome measures, and statistical analyses were used. The confounder criterion was not applicable to fifteen of the twenty cross-sectional studies^(33–36,39–41,44–51)^ because of its descriptive characteristics, without comparison groups. Therefore, the ‘5’ and ‘6’ checklist items were not considered in the calculation of the percentage for these studies. The included cohort study^(52)^ achieved high scores despite the limitations in the strategy used to address incomplete follow-up.

**Table 5.** Quality of cross-sectional studies according to the Joanna Briggs Institute Critical Appraisal Checklist, 2025

Studies	1. Were the criteria for inclusion in the sample clearly defined?	2. Were the study subjects and the setting described in detail?	3. Was the exposure measured in a valid and reliable way?	4. Were objective, standard criteria used for measurement of the condition?	5. Were confounding factors identified?	6. Were strategies to deal with confounding factors stated?	7. Were the outcomes measured in a valid and reliable way?	8. Was appropriate statistical analysis used?	Percentage (%)
Previdelli et al. (2010)	No	Yes	Yes	Yes	Yes	Yes	Yes	Yes	87.5
Canella et al. (2011)	Yes	Yes	Yes	Yes	NA	NA	Yes	Yes	100
Mesquita & Mesquita (2013)	Yes	Yes	Yes	Yes	NA	NA	No	No	66.6
Lanci & Matsumoto (2013)	No	Yes	No	Yes	NA	NA	Yes	No	50
Pereira et al. (2014)	Yes	No	Yes	Yes	NA	NA	Yes	No	66.6
Cunha & Barbosa (2014)	No	No	Yes	Yes	NA	NA	No	No	33.3
Duarte et al. (2015)	No	No	Yes	Yes	NA	NA	Yes	No	50
Batista et al. (2015)	Yes	Yes	Yes	Yes	NA	NA	No	No	66.6
Bezerra (2015)	Yes	Yes	Yes	Yes	Yes	Yes	Yes	Yes	100
Oro & Hautrive (2016)	No	No	Yes	Yes	NA	NA	Yes	No	50
Ferreira (2016)	No	No	No	Yes	NA	NA	No	No	16.6
Bortolozo et al. (2016)	Yes	Yes	Yes	Yes	NA	NA	No	Yes	83.3
Bezerra et al. (2017)	Yes	Yes	Yes	Yes	Yes	Yes	Yes	Yes	100
Salvetti & Possa (2017)	No	No	Yes	Yes	NA	NA	Yes	Yes	66.6
Hartmann et al. (2019)	No	No	Yes	Yes	NA	NA	No	No	33.3
Albuquerque & Lima (2020)	No	No	Yes	Yes	NA	NA	No	No	33.3
Guilherme et al. (2020)	Yes	Yes	Yes	Yes	NA	NA	Yes	Yes	100
Souza (2020)	Yes	Yes	Yes	Yes	Yes	Yes	Yes	Yes	100
Costa et al. (2022)	Yes	Yes	Yes	Yes	Yes	Yes	Yes	Yes	100
Bergamin (2022)	No	No	Yes	Yes	NA	NA	No	No	33.3

**Table 6.** Quality of cohort studies according to the Joanna Briggs Institute Critical Appraisal Checklist, 2025

Study	1. Were the two groups similar and recruited from the same population?	2. Were the exposures measured similarly to assign people to both exposed and unexposed groups?	3. Was the exposure measured in a valid and reliable way?	4. Were confounding factors identified?	5. Were strategies to deal with confounding factors stated?	6. Were the groups/participants free of the outcome at the start of the study (or at the moment of exposure)?	7. Were the outcomes measured in a valid and reliable way?	8. Was the follow-up time reported and sufficient to be long enough for outcomes to occur?	9. Was follow-up complete, and if not, were the reasons for loss to follow-up described and explored?	10. Were strategies to address incomplete follow-up utilised?	11. Was appropriate statistical analysis used?	Percentage (%)
Torres et al. (2020)	Yes	Yes	Yes	Yes	Yes	NA	Yes	Yes	Yes	No	Yes	90

## Discussion

The studies included in the review that analysed the Brazilian WFP were conducted in different states and regions of the country and showed that in general the programme did not achieve its nutritional guidelines. Considering the programme’s wide coverage and that the average annual growth rate is about 8.7% in the number of workers benefiting and 14.5% in the number of companies participating in the WFP,^(14)^ this scenario may threaten the food and nutritional security of the beneficiaries over time. The inadequacy of meals offered or consumed in some nutritional aspects, and the nutritional status of the workers may be aligned with the changes in the Brazilian dietary pattern that have occurred in recent years, with a reduction in the consumption of traditional foods such as rice and beans, a high consumption of ultra-processed foods, generally rich in sodium, sugar, and calories, and an increase in the prevalence of obesity;^(55–57)^ however, it would be expected that this trend would be attenuated among individuals who are beneficiaries of the programme.

The amount of sodium found in the lunches evaluated is close to the amount recommended by the WHO for the whole day (<2000 mg),^(58)^ showing that just one meal is likely to meet almost the entire daily sodium requirement. However, the high amount of sodium verified in the of WFP meals is not different from the high sodium consumption of the Brazilian population.^(59)^ This finding is worrisome because excessive sodium consumption is a risk factor for hypertension, which can affect the health of workers and cause temporary disability.^(60)^

The offer of artificial juices, soft drinks, sweets as desserts, fried foods, and fatty meats, identified in some studies, confirms the results that in some companies the lunches served to workers were inadequate, at least in terms of nutritional aspects.^(36,47,51)^ Therefore, these results showed that some critical items that contribute to increase caloric intake and reduce the quality of meals are present in the evaluated menu. In addition, some studies have shown a lack of fruits and vegetables on the daily menu.^(36,47,48)^ It is interesting to note that the average meal quality index obtained in one of the included studies^(48)^ was close to the findings of another study,^(20)^ classified as a ‘meal in need of improvement’, which shows that the meals served by these companies are not in line with the recommendations for a healthy diet.

The excessive offer of protein on the menu may reflect the reality of companies that offer two meat options every day,^(45)^ as well as two food preparations with meat in their composition and one preparation with egg (fried egg, omelette, or scrambled egg).^(41)^ In addition, these preparations are not always portioned, and the worker can serve himself or herself in all options without control.^(40)^ The consumption of animal protein is generally associated with a higher intake of saturated fat.^(60)^ Although the intake of saturated fat was within the guidelines, the intake of polyunsaturated fat was below the recommended level, and the cholesterol intake was above the recommended level, nutrients that are not included in the recommendations of the programme but that may indicate the low quality of the meals offered.^(46)^

When analysing the WFP in the city of São Paulo, similar results were found when they observed that the intake of polyunsaturated fats was inadequate.^(20)^ Epidemiological studies have shown that some nutrients are associated with noncommunicable diseases, highlighting the excessive consumption of fats such as cholesterol.^(58,61–63)^ Therefore, the lipid content of the meals offered must remain within the recommended range, using more vegetable oils rich in polyunsaturated fatty acids and reducing the supply of foods of animal origin rich in cholesterol.^(64)^

Concerning carbohydrate provision, some studies have also found an inadequate supply in their assessments.^(65–68)^ This result shows the importance of increasing the supply of fruits and vegetables in the meals offered by the companies registered in the programme, since in addition to increasing the carbohydrate supply, increasing the variety of the menu would also increase, contribute to reducing the energy density of the meals offered.

The supply of fibre in the meals offered to workers was related to the relevant consumption of beans, which contributed on average 64.31% of the total fibre observed in the assessment.^(48)^ In addition to beans, a supply of vegetables was observed in both appetisers and side dishes, increasing the amount of this nutrient in the meals offered to workers.^(69)^

In terms of nutritional status, the high prevalence of overweight and obesity among WFP workers may reflect the quality of the food offered by these companies and the limitations in the implementation of the programme as well as the nutritional status of the Brazilian population in general.^(32,34,35,37,45,50)^ The research that found the lack of knowledge of managers and nutritionists about the objectives of the programme^(51)^ reinforces the idea that the WFP seems to represent a model of financial subsidy for companies rather than a programme focused on workers and their health and nutritional conditions.^(70,71)^ It is important to emphasise that all companies should have a nutritionist as a technical manager due to their essential role not only in menu planning but also in monitoring the nutritional status and incidence of noncommunicable diseases among workers.

Studies comparing WFP beneficiaries and non-beneficiaries found a higher prevalence of obesity and greater cardiovascular risk among WFP beneficiaries.^(38,42,43)^ However, the only nutrients that were consumed more by WFP beneficiaries than by non-WFP beneficiaries were carbohydrates and fibre at lunch among men.^(42)^ This fact may illustrate the limitations of the approach of Normative Ordinance No. 66/2006,^(15)^ which is based, although not exclusively, on nutrients. In fact, it is important to evaluate the quality of the food offered/consumed by workers since not only the amount of nutrients but also the food processing may be a determining factor for excessive weight gain.

The study that evaluated food consumption among workers using the NOVA classification found similar results related to the contribution of ultra-processed foods (19.7%) in the Brazilian Household Budget Survey.^(56)^ In addition, there was a greater influence of sensory appeal, price and convenience factors when evaluating motivations for food choices.^(37)^ It is known that ultra-processed foods are rich in food additives, sugar, saturated fat and salt, making them highly palatable, as well as being affordable and very convenient.^(72)^ Awareness of these motivations can help to understand why companies, even those registered with the WFP, find it difficult to motivate their employees to eat an adequate and healthy diet. In practice, even if the planned menu complies with the Normative Ordinance No. 6/2006,^(15)^ it is necessary to limit the supply of ultra-processed foods in the company’s menu.

It is also important to compare workers from different sectors of the company.^(50)^ When evaluating the consumption of meals provided by the company, energy and lipid consumption differed according to the sector of activity, being greater for workers in the production sector, who also had a higher energy expenditure, than for workers in the administrative sector. When planning menus, it is important to take these specificities into account to avoid under- or overestimating the nutritional needs of workers in different sectors.

This review highlights the paucity of studies that have evaluated smaller and micro companies and other sectors, such as services and trade, which cover a large proportion of the programme’s target workers. The WFP continues to focus mainly on companies with a larger number of workers because, in its current form, companies that do not have a specific tax regime under the programme’s rules do not receive tax benefits for joining the programme, which creates a disincentive for their enrolment and harms their workers.^(14)^

Moreover, although not the most common modality in WFP, the provision or consumption of meals at the workplace was the only one evaluated in the studies included in this review.^(16)^ Despite being the most common modalities, meals/food vouchers and food baskets have been less studied in food and nutrition research, possibly due to the difficulty of operationalising the guidelines of the Normative Ordinance No. 66/2006.^(15)^

According to this regulation, each meal should simulate a day’s diet, ignoring the differences in meal profiles. It does not make sense to expect breakfast and lunch to have the same nutrient distribution, since they are meals that contain foods with different compositions, such as dairy products, which are rich in calcium; bread, which is rich in carbohydrates, for breakfast; and rice and beans, which are rich in protein and fibre, for lunch. In addition, it is difficult to calculate the value of meals/food vouchers, and important criteria, such as limiting purchases to stores that predominantly sell ultra-processed products, are lacking to qualify the programme for adequate and healthy nutrition promotion. Currently, the lack of nutritional guidelines for the programme makes this scenario even more worrying, highlighting the need to align the recommendations with the country’s main tool for food and nutrition education, the Brazilian Dietary Guidelines.^(73)^

It is important to emphasise that the interpretation of the results of the studies included in this review was made in the light of the guidelines of the Normative Ordinance No. 66/2006,^(15)^ which was in force at the time of data collection. Studies carried out before this period evaluated the adequacy of the programme in a different way, considering that its nutritional guidelines aimed to overcome energy deficiency in adults in the past due to the high prevalence of malnutrition in Brazil.^(74)^

Moreover, the low methodological quality found in some studies may reflect the difficulty of conducting this type of research in the workplace. As the WFP is a public policy implemented in the private sector, it can be difficult to access companies and obtain compliance with these surveys. In addition, the lack of technical preparation sheets in some food and nutrition units makes it difficult to evaluate the food provided according to the nutritional guidelines established by the programme.^(51)^

In the context of the global scenario, government policies that specifically address workers’ health and nutrition are scarce. For the most part, their policies include interventions that target only workers in the formal sector, as is the case with the WFP. There are also countries that provide free tools, resources, and information to support the development of workplace health and wellness programmes. For example, the toolkit in Australia includes training on how to promote healthy habits in the workplace, such as encouraging the inclusion of healthy food and drink options in meetings, vending machines, canteens, or workplace kiosks.^(3)^ Another example is programmes that develop food and nutrition education interventions aimed at chronic disease prevention. This is the case of the Choose Healthy Now project in Hawaii, United States, which is being implemented in snack and beverage vending machines, including workplaces. This project aims to educate consumers about food consumption by focusing on labels and developing a rating system that takes into account the nutritional standards established by the state’s health guidelines – green (healthiest), yellow (intermediate), and red (least healthy).^(1)^

Despite the limitations revealed by the results of this review, the WFP is a programme that has great potential because of its nationwide scope due to the legislation that applies to the entire country and because it offers the possibility of different benefit modalities, unlike other world programmes. On the other hand, their recommendations did not define criteria aimed at limiting the supply and advertising of products with an unbalanced nutritional composition, such as the Food Service Guidelines, based on the Dietary Guidelines for Americans, for the purchase of food to be offered in the workplace.^(2,75–77)^

This systematic review had some limitations. A meta-analysis of the data was not possible due to the heterogeneity of the outcome and exposure measures of the included articles. The small number of longitudinal studies could be a limitation to ensure the association between the quality of the nutrient composition of foods/meals and health-related measures of workers. In addition, this review has limitations related to the difficulty in finding recent documents and reports from governmental or non-governmental associations to strengthen the discussion of the results. Outdated government websites and the lack of updated regulations for the programme reflect how this debate has been put on the back burner in Brazil.

Some of the strengths of this review include our systematic approach based on the PRISMA guidelines, the peer review, the assessment of study quality using the Joanna Briggs Institute’s critical appraisal tool, and the inclusion of twenty-one studies covering a range of states and regions and a large number of companies and workers evaluated. In this way, this systematic review provides a perspective on the current state of the WFP in Brazil.

Our findings indicate that the quality and nutritional value of the food provided to and consumed by workers falls short of the required standards and, in some cases, does not promote healthy diets. Also, despite the technical supervision of nutritionists in some companies, the expected nutritional quality is not always found, which shows that the nutritional guidelines are not always considered. The WFP has the potential to be a food and nutrition security programme, but our review found that despite its long history, the programme has made little progress. The evidence suggests that the WFP needs to be reformulated in the face of existing challenges, defining new specific guidelines for each modality of implementation, taking into account the different sectors of the companies, and making it a programme that contributes to strengthening the realisation of the human right to adequate food.

Future research should focus on the meal/food vouchers and food baskets to better understand how they affect the health of workers benefiting from the WFP and to support strategies to improve them. There is also a need to study the food and nutritional conditions of informal workers in the country, as their right to quality food is not guaranteed. Improving national public policies in this area can help to address this issue.

### Conclusion

In conclusion, the current review provides support for understanding the food and nutrition context of the Brazilian workers’ WFP benefiting, pointing to evidence that the programme does not fully meet the required nutritional guidelines, and consequently, does not promote an adequate and healthy diet. This shows how much attention the food and nutrition of workers deserves. The findings of this review could strengthen the dialogue between policymakers and civil society and support the reformulation and strengthening of the programme in Brazil.

## Supporting information

Albuquerque et al. supplementary material Albuquerque et al. supplementary material

## Supplementary material

The supplementary material for this article can be found at https://doi.org/10.1017/jns.2025.20.
