# Supplementary material for: The nutritional quality of the meals and foods provided to beneficiaries of the Brazilian Worker’s Food Program: a systematic review
Source: J Nutr Sci. 2025 Mar 22;14:e31. doi: 10.1017/jns.2025.20 (PMC12034498; doi:10.1017/jns.2025.20)
Supplement: Albuquerque et al. supplementary material [file S2048679025000205sup001.docx]

Supplementary Material. Search strategy carried out in each database.

Pubmed


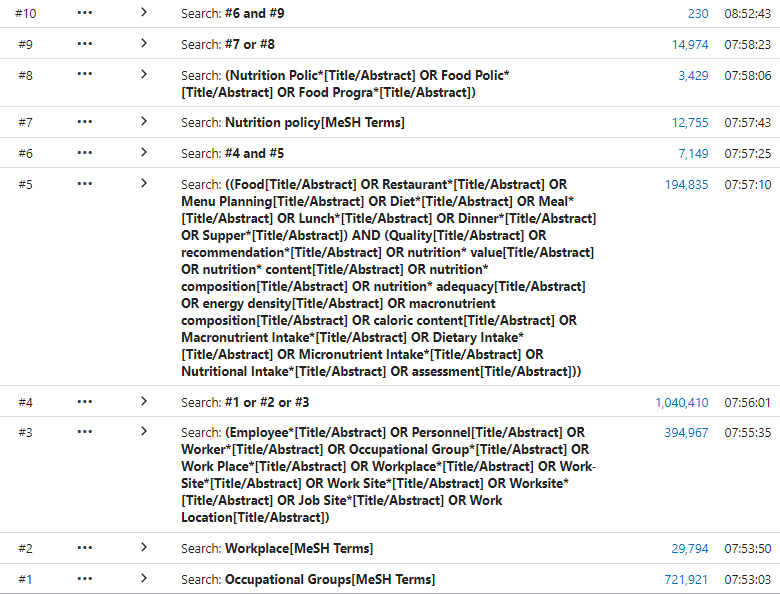


Scopus

( ( ( INDEXTERMS ( "Occupational Groups" ) ) OR ( INDEXTERMS ( "Workplace" ) ) OR ( TITLE-ABS-KEY ( "Employee*" OR "Personnel" OR "Worker*" OR "Occupational Group*" OR "Work Place*" OR "Workplace*" OR "Work-Site*" OR "Work Site*" OR "Worksite*" OR "Job Site*" OR "Work Location*" ) ) ) AND ( ( TITLE-ABS-KEY ( "food" OR "restaurant*" OR "menu planning" OR "diet*" OR "meal*" OR "lunch*" OR "dinner*" OR "supper*" ) AND TITLE-ABS-KEY ( "quality" OR "recommendation*" OR "nutrition* value" OR "nutrition* content" OR "nutrition* composition" OR "nutrition* adequacy" OR "energy density" OR "macronutrient composition" OR "caloric content" OR "macronutrient intake*" OR "dietary intake*" OR "micronutrient intake*" OR "nutritional intake*" OR "assessment" ) ) ) ) AND ( ( INDEXTERMS ( "nutrition policy" ) ) OR ( TITLE-ABS-KEY ( "nutrition polic*" OR "food polic*" OR "food progra*" ) ) )

Embase


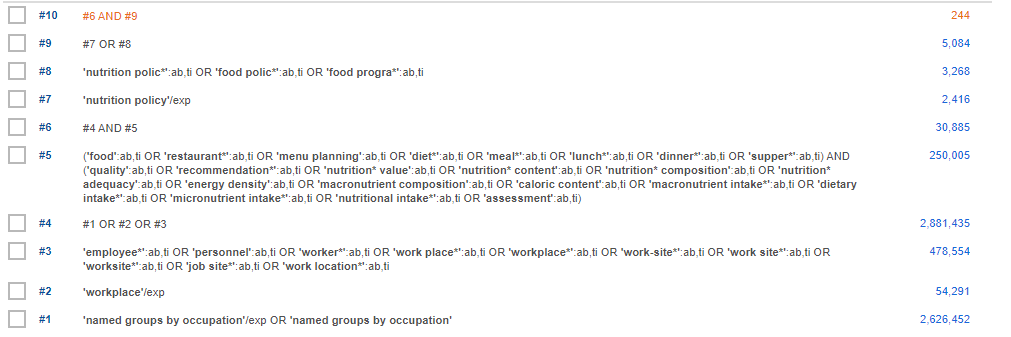


Lilacs and Scielo

(("WORKER" or "TRABALHADOR" or "WORKPLACE" or "LOCAL DE TRABALHO")) AND (("FOOD SERVICE" or "SERVICO DE ALIMENTACAO" or "MENU" or "CARDAPIO" or "MEAL" or "REFEICAO" or "LUNCH" or "ALMOCO" or "RESTAURANT" or "RESTAURANTE" or "Nutritional Quality" or "Qualidade nutricional") )

Web of Science


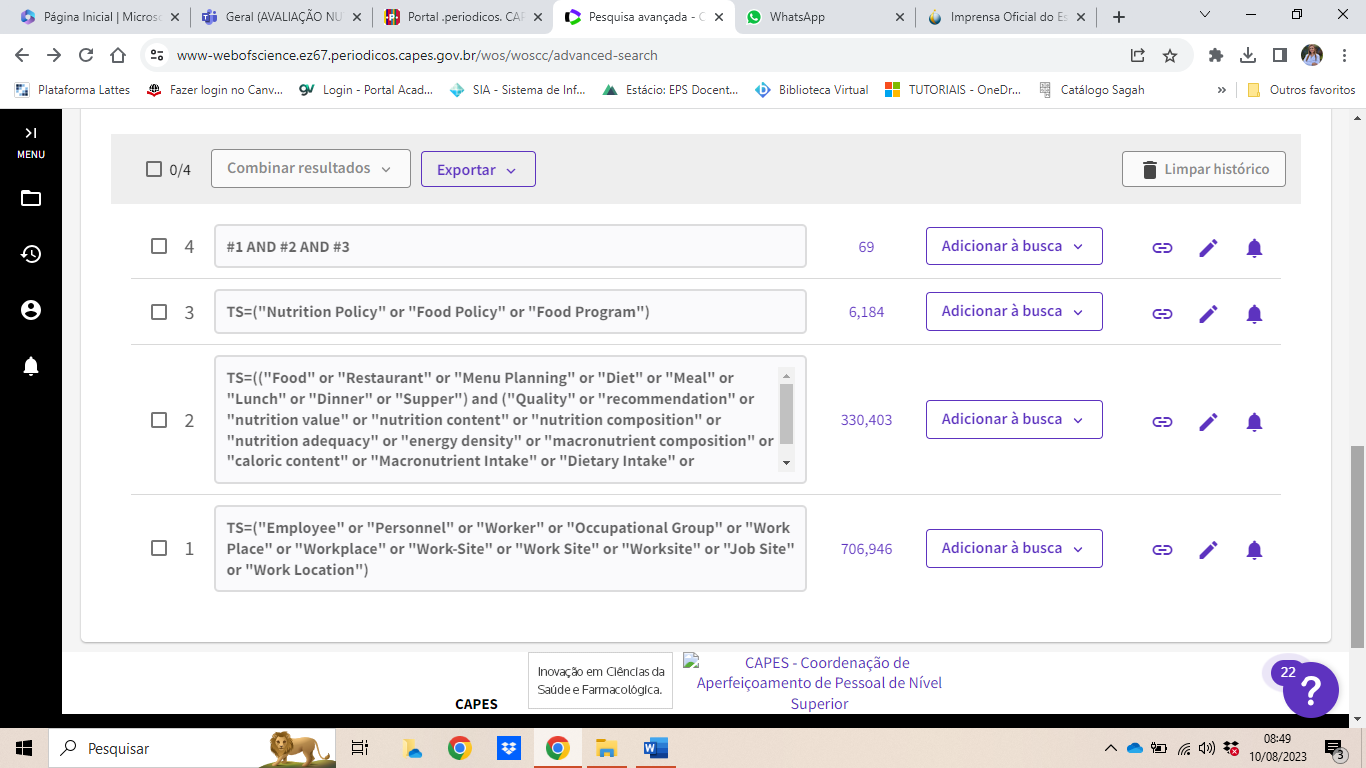


Google scholar

(meals OR lunch OR menu OR workplace OR worker "worker food program")
